# Sulindac activates NF-κB signaling in colon cancer cells

**DOI:** 10.1186/1478-811X-11-73

**Published:** 2013-10-01

**Authors:** Dessislava Mladenova, Laurent Pangon, Nicola Currey, Irvin Ng, Elizabeth A Musgrove, Shane T Grey, Maija RJ Kohonen-Corish

**Affiliations:** 1 Kinghorn Cancer Centre, Garvan Institute of Medical Research, 370 Victoria St, Darlinghurst, Sydney, NSW 2010, Australia; 2 St. Vincent’s Clinical School, Faculty of Medicine, UNSW, Sydney, NSW, Australia; 3 Immunology Research Program, Garvan Institute of Medical Research, Sydney, NSW, Australia; 4 School of Medicine, University of Western Sydney, Sydney, Australia

**Keywords:** Pro-inflammatory cytokines, NF-kB, AP-1, IL-8, A20, Colon epithelial cells

## Abstract

**Background:**

The non-steroidal anti-inflammatory drug (NSAID) sulindac has shown efficacy in preventing colorectal cancer. This potent anti-tumorigenic effect is mediated through multiple cellular pathways but is also accompanied by gastrointestinal side effects, such as colon inflammation. We have recently shown that sulindac can cause up-regulation of pro-inflammatory factors in the mouse colon mucosa. The aim of this study was to determine the signaling pathways that mediate the transcriptional activation of pro-inflammatory cytokines in colon cancer epithelial cells treated with sulindac sulfide.

**Results:**

We found that sulindac sulfide increased NF-κB signaling in HCT-15, HCT116, SW480 and SW620 cells, although the level of induction varied between cell lines. The drug caused a decrease in IκBα levels and an increase of p65(RelA) binding to the NF-κB DNA response element. It induced expression of IL-8, ICAM1 and A20, which was inhibited by the NF-κB inhibitor PDTC. Sulindac sulfide also induced activation of the AP-1 transcription factor, which co-operated with NF-κB in up-regulating IL-8. Up-regulation of NF-κB genes was most prominent in conditions where only a subset of cells was undergoing apoptosis. In TNFα stimulated conditions the drug treatment inhibited phosphorylation on IκBα (Ser 32) which is consistent with previous studies and indicates that sulindac sulfide can inhibit TNFα-induced NF-κB activation. Sulindac-induced upregulation of NF-κB target genes occurred early in the proximal colon of mice given a diet containing sulindac for one week.

**Conclusions:**

This study shows for the first time that sulindac sulfide can induce pro-inflammatory NF-κB and AP-1 signaling as well as apoptosis in the same experimental conditions. Therefore, these results provide insights into the effect of sulindac on pro-inflammatory signaling pathways, as well as contribute to a better understanding of the mechanism of sulindac-induced gastrointestinal side effects.

## Background

NSAIDs, including sulindac, have shown promising potential in colon cancer chemoprevention [1]. The chemopreventive potential of sulindac and other NSAIDs was initially attributed to COX inhibition, however, it is now recognized that the anti-proliferative and anti-inflammatory effects of sulindac can also be mediated through non-COX targets [2-5]. Sulindac sulfide is the pharmacologically active metabolite of sulindac, and is 5000-fold more potent in inhibiting COX activity than the sulfone metabolite. It can also induce apoptosis in colon cancer cells at concentrations 4–5 times lower than those of the sulfone metabolite [6].

Long-term NSAID use is associated with gastrointestinal and cardiovascular side effects, including colon mucosal inflammation, which has also been reported in mice [7-9]. We have recently shown that apart from chemoprevention, long-term sulindac administration induces inflammatory lesions in the mouse colon, which show up-regulation of pro-inflammatory factors such as MIP-2 (the mouse homologue of IL-8) [9] and can progress to cancer in mice that are deficient for tumor suppressor genes. The mechanism by which sulindac can up-regulate pro-inflammatory genes is not known, but some of these genes are regulated by NF-κB, whose activity is central to orchestrating the immune response [10]. This prompted us to investigate the molecular effects of sulindac sulfide on the NF-κB pathway *in vitro*.

NF-κB is the collective name for a family of transcription factors and is a major regulator of processes such as inflammation, cell survival and apoptosis. The NF-κB pathway can be activated by a large variety of factors, including cytokines and stress stimuli and NF-κB activation is central to the pathogenesis of chronic inflammatory disorders such as inflammatory bowel disease [11]. Cytokines and growth factors can also induce the activator protein-1 (AP-1) transcription factor, which regulates genes involved in numerous tumor-promoting functions as well as inflammatory processes [12].

NF-κB dimers function as a transcription factor in the nucleus and are sequestered in an inactive form in the cytoplasm, bound to Inhibitor of kappa B proteins (IκB), most often IκBα. Upon stimulation by pro-inflammatory cytokines, such as TNFα and IL-1, I kappa B kinase (IKK) is activated. IKK phosphorylates IκBα, which is then degraded by the proteasome, allowing translocation of the NF-κB dimers to the nucleus. NF-κB signaling can be modulated by NSAIDs in experimental models but the exact mechanism is poorly understood [13,14]. Sulindac or its derivatives, sulindac sulfone and sulindac sulfide, were found to inhibit NF-κB activation in stimulated COS, human leukemic and human embryonic kidney cell lines [14,15]. However, sulindac and sulindac sulfide had no effect on NF-κB driven reporter gene expression in non-stimulated cells (basal levels of NF-κB activity) [15,16]. Subsequent studies in colon cancer cells found that aspirin, sulindac and sulindac sulfone inhibit NF-κB-dependent transcriptional activity and cause apoptosis, but this was shown to involve initial NF-κB pathway activation through IκBα degradation and NF-κB nuclear translocation [17-19]. Therefore the effect of sulindac and its derivatives on NF-κB signaling may vary depending on the experimental conditions.

The aim of this study was to determine the signaling pathways leading to sulindac sulfide induced upregulation of IL-8 and other pro-inflammatory mediators in the colon. IL-8 was chosen because of the strong effect of sulindac on inducing MIP-2 (the mouse homologue of IL-8) in the mouse colon mucosa in our previous study, [9] while ICAM1 is considered to be a classic NF-κB target [19]. A20 was chosen for this study because it is an early response NF-κB target gene, which is not known to be targeted by any other transcription factor. NF-κB activation is necessary for A20 transcription as IKK deficiency abolishes TNFα-induced A20 transcription [20,21]. We have used COX-2 non-expressing or low-expressing cell lines in order to study COX-independent effects of sulindac sulfide. We provide evidence that sulindac sulfide can activate both NF-κB and AP-1 signaling pathways in the colon mucosa leading to upregulation of IL-8.

## Results

### Sulindac sulfide induces up-regulation of NF-κB target genes and concurrently induces cell death in HCT-15 colon cancer cells

**Figure 1:**
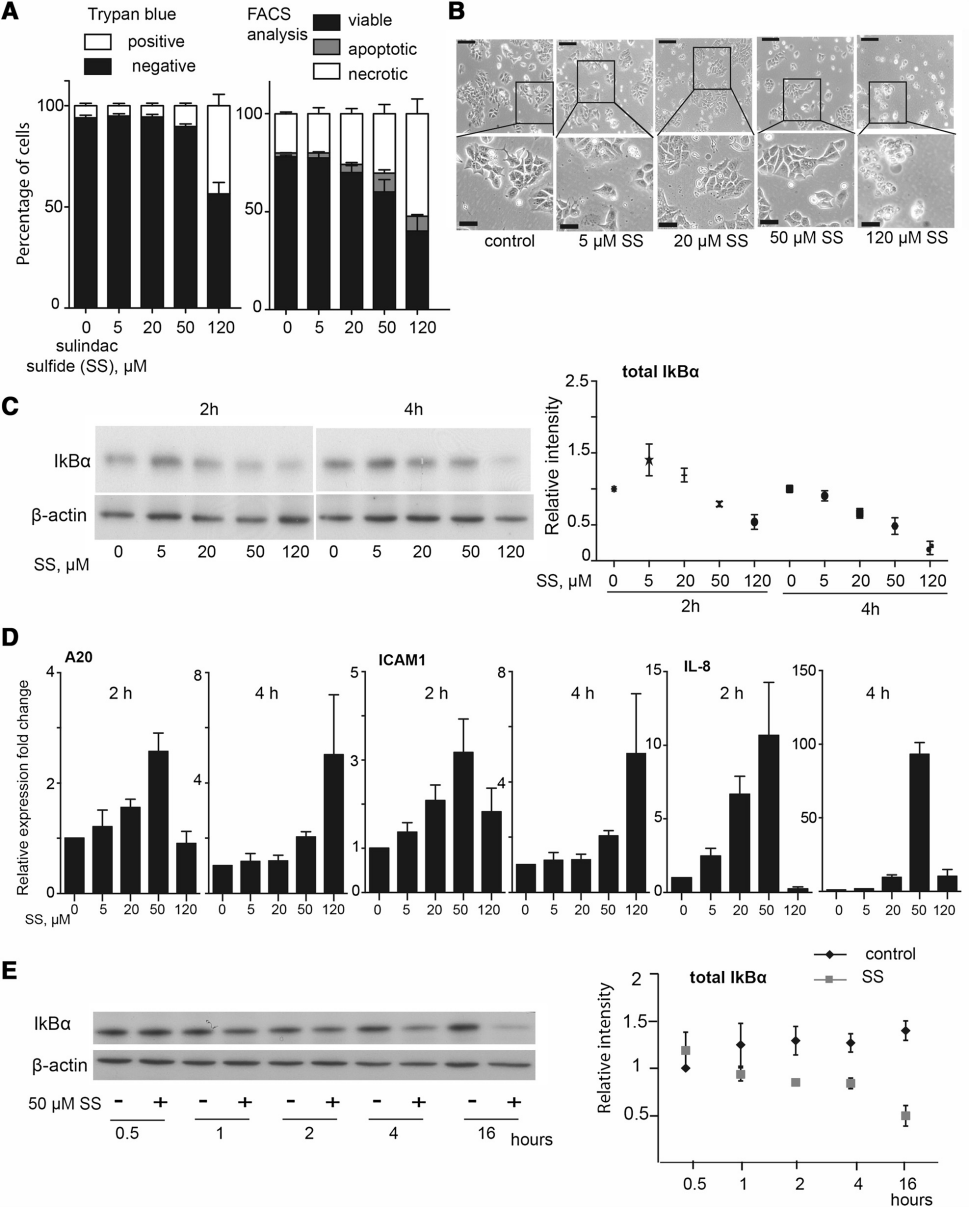
**Sulindac sulfide induces activation of the NF-κB pathway and increases cell death. (A)** Quantification of apoptosis and necrosis of sulindac sulfide (SS) treated cells (4 hours), assessed by trypan blue exclusion assay or FACS analysis of Annexin-V-Fluos/PI stained cells. Bars represent mean percentage of cells in each population ± SEM (n = 3-4). **(B)** Photomicrographs of HCT-15 cells treated with sulindac sulfide for 8 hours (phase-contrast, scale bars 60 μm and insert 24 μm). **(C)** HCT-15 cells were treated with the indicated doses of sulindac sulfide or the control DMSO (equivalent to the highest dose SS) for 2 and 4 hours, after which lysates were collected and western blot analysis was performed for IκBα and the loading control β-actin. Densitometry of IκBα levels normalized to β-actin. Error bars represent SEM (n = 3-4). **(D)** qPCR analysis for A20, ICAM1 and IL-8 mRNA expression. HCT-15 cells were treated with the indicated concentrations of SS or the control. Gene expression was normalized to the house-keeping gene GAPDH. The data are presented as fold change ± SEM (from 3 independent experiments). **(E)** Western blot for IκBα levels in cells treated with SS or the control for the indicated time points. Densitometry of IκBα levels normalized to β-actin. Error bars represent SEM.

As apoptosis induction is one of the well-established anti-tumorigenic mechanisms of sulindac sulfide [6] we first established a concentration that induces apoptosis in HCT-15 cells. Sulindac sulfide treatment induced concentration-dependent cell death (Figure 1A). A concentration of 50 μM significantly increased apoptotic cell death (4.7 fold ± 0.6 SEM, P ≤ 0.01) while leaving the majority of the cell population viable at 4 h. The higher concentration, 120 μM sulindac sulfide also induced a significant increase in necrosis (2.6 fold ± 0.4 SEM, P ≤ 0.02) (Figure 1A). In addition the 120 μM concentration caused a change in morphology - cell rounding and a swollen appearance, indicating toxicity with higher concentrations (Figure 1B).

It was previously shown that sulindac and sulindac sulfone decrease the protein level of NF-κB inhibitor IκBα in colon cancer cells within 2–5 hours [19]. We next assessed the effect of sulindac sulfide on IκBα. Treatment of HCT-15 cells with 50 μM and 120 μM sulindac sulfide decreased IκBα protein levels after 2 hours and 20 μM, 50 μM and 120 μM after 4 hours of treatment (Figure 1C). The decrease in IkBα was accompanied by an increase in mRNA expression of NF-κB target genes A20, ICAM1 and IL-8, which was more pronounced with 50 or 120 μM sulindac sulfide (Figure 1D). Thus the 50 μM concentration of sulindac sulfide could trigger apoptosis and pro-inflammatory gene up-regulation in the same experimental conditions.

In order to determine if this activation was transient or sustained, we studied the kinetics of IκBα degradation in cells treated with 50 μM sulindac sulfide for 0.5, 1, 2, 4 and 16 hours. We observed a significant decrease of IκBα protein levels 2 hours post treatment (P < 0.05) and this was sustained until the conclusion of the experiment at 16 hours (Figure 1E). This was not due to decreased transcription as 50 μM sulindac sulfide actually increased IκBα mRNA transcripts by 3.3 fold (± 1.14 SEM) 4 hours post-treatment compared to control-treated cells, which is consistent with NF-κB pathway activation where increased transcription of IκBα is an early response.

### Sulindac sulfide-induced pro-inflammatory gene up-regulation is dependent on NF-κB activity but is not mediated by apoptosis

**Figure 2:**
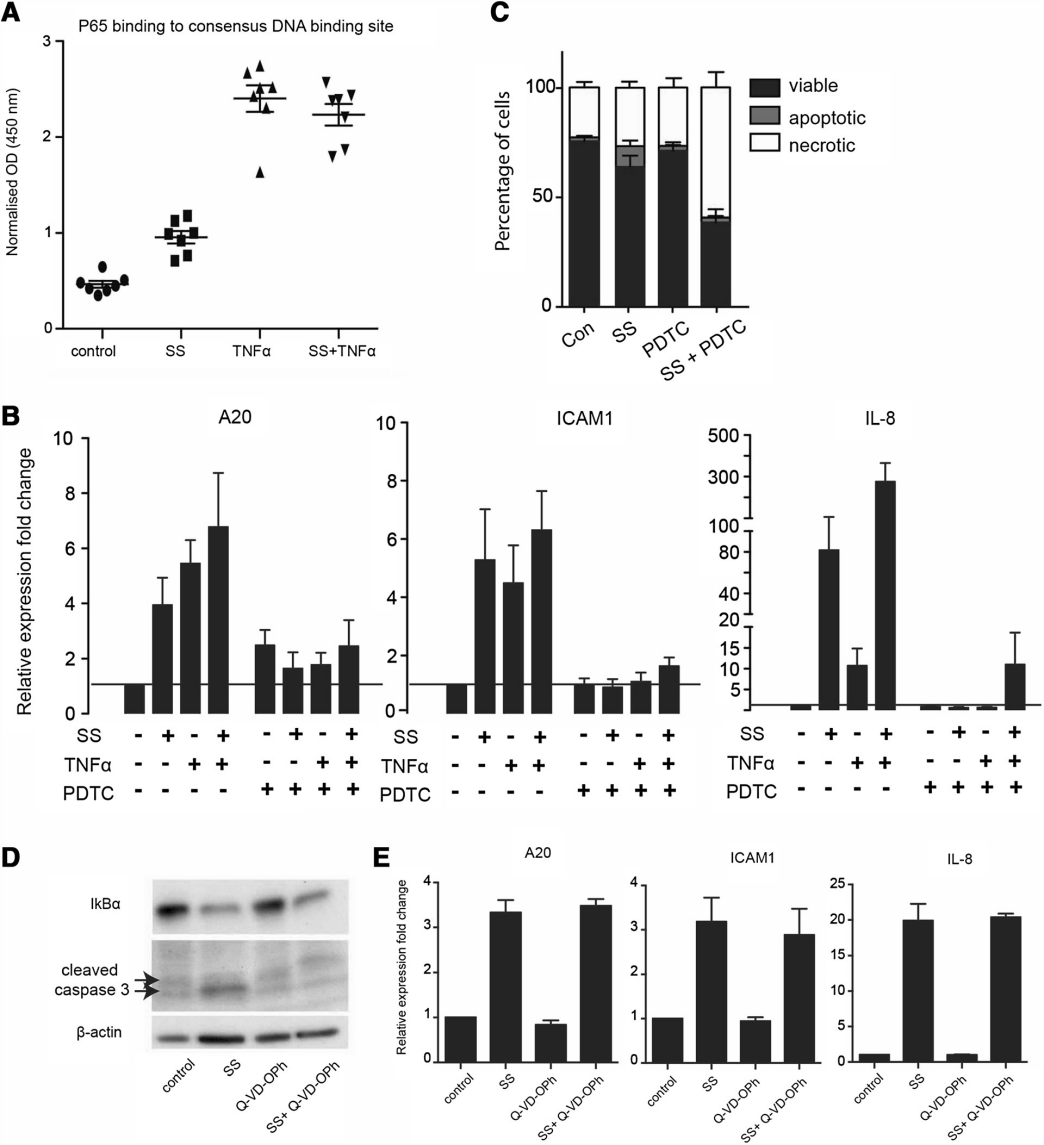
**Sulindac sulfide-induced pro-inflammatory gene up-regulation is dependent on NF-κB activity but is not mediated by apoptosis. (A)** Colorimetric assay of p65 DNA binding activity to NF-κB response element in nuclear lysates of HCT-15 cells. Cells were treated with the control or SS for 4 hours and where indicated 10 ng/ml TNFα was added for the last 40 minutes before cell lysis. The DNA-binding activity of p65 is expressed as the optical density at 450 nm. Error bars indicate SEM. **(B)** qPCR analysis for A20, IL-8 and ICAM-1 mRNA expression levels. HCT-15 cells were pretreated with or without 50 μM PDTC for 1.5 hours, followed by treatment with the control (DMSO), 50 μM sulindac sulfide (SS), 10 ng/ml TNFα or both in combination for 4 hours. Bars represent fold change of relative gene expression, normalized to the house-keeping gene GAPDH. Mean values ± SEM (from 3 to 4 independent experiments). **(C)** Quantification of apoptosis and necrosis of cells treated with 50 μM SS, PDTC or both compounds in combination for 4 hours, assessed by FACS analysis of Annexin-V-Fluos/PI stained cells. Bars represent mean percentage of cells in each population ± SEM (n = 3). **(D)** HCT-15 cells were pre-treated with 20 μM pan-caspase inhibitor Q-VD-OPh or the control DMSO for 1 hour and then stimulated with 50 μM sulindac sulfide (SS) or the vehicle DMSO (control) for 2 hours A western blot analysis for cleaved caspase 3 (17/19 kDa) and the house-keeping gene β-actin. **(E)** qPCR analysis for A20, ICAM1 and IL-8 in cells treated with the indicated concentrations of SS in the presence or absence of Q-VD-Oph for 2 hours. The data are presented as fold change ± SEM (from 3 independent experiments).

NF-κB is most commonly composed of p50 and p65(RelA) heterodimers, of which only p65 has transactivation potential [22]. We tested whether sulindac sulfide increases the binding of p65 to the NF-κB DNA response element. HCT-15 cells were treated with sulindac sulfide and/or TNFα and nuclear lysates were prepared. A colorimetric p65 transcription factor assay was used to assess the amount of nuclear p65 bound to the consensus NF-κB response element immobilized on the assay plate (Figure 2A). Both sulindac sulfide alone and TNFα alone significantly increased p65 binding to DNA (P < 0.01).

In order to test whether sulindac sulfide-induced pro-inflammatory gene up-regulation is dependent on NF-κB activity, we treated cells with the NF-κB specific inhibitor PDTC [23]. Pre-treatment of cells with 50 μM PDTC effectively inhibited both TNFα-induced and sulindac sulfide-induced up-regulation of the NF-κB target genes A20, ICAM-1 and IL-8 (Figure 2B). Concurrent treatment of cells with PDTC and sulindac sulfide also reduced the proportion of viable cells (Figure 2C). Thus PDTC potentiated sulindac sulfide-induced cancer cell death.

Cells undergoing cell death can release pro-inflammatory molecules such as high mobility group box 1 protein (HMGB1) that can induce NF-κB signaling cascade [24]. Therefore, we next tested whether sulindac sulfide-induced apoptotic response is involved in NF-κB activation. In order to inhibit sulindac sulfide-induced apoptosis, we pre-treated cells with the irreversible caspase inhibitor Q-VD-OPh, a broad-spectrum caspase inhibitor with very low cytotoxicity which is also known to inhibit HMGB1 release [25]. We assessed NF-κB activation by qPCR for the NF-κB target genes A20, ICAM-1 and IL-8 in the presence or absence of the caspase inhibitor. Q-VD-OPh effectively inhibited sulindac sulfide-induced apoptosis, assessed by western blot analysis for cleaved caspase 3 (Figure 2D). NF-κB target genes were significantly up-regulated in cells co-treated with sulindac sulfide and Q-VD-OPh compared to control treated cells and cells treated with the caspase inhibitor alone (Figure 2E). Therefore these data suggest that sulindac sulfide-induced NF-κB activation is not mediated by the apoptotic response, triggered by the drug.

### Sulindac treatment of mice induces pro-inflammatory genes within one week

**Figure 3:**
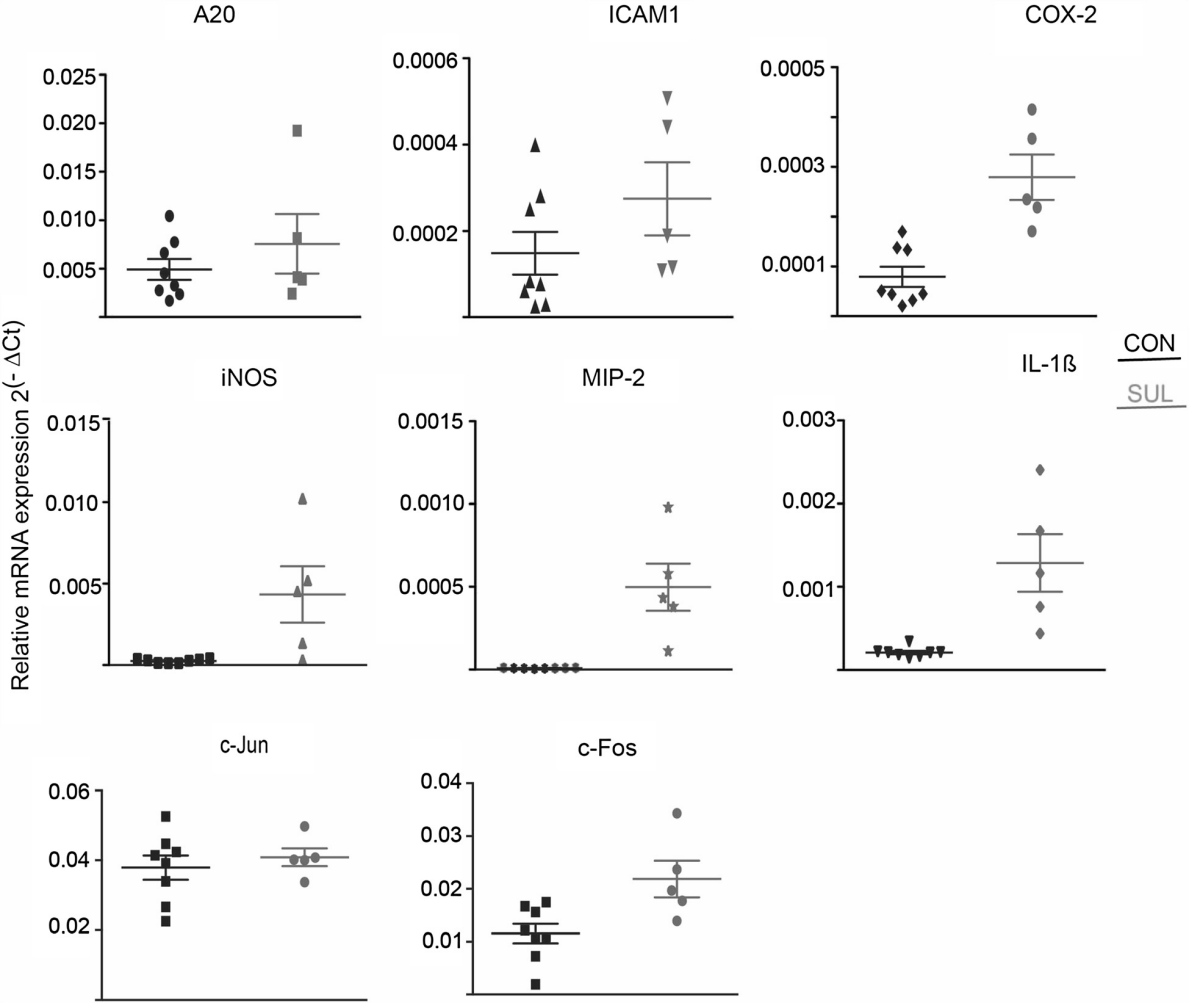
**Sulindac feed modulates the expression of pro-inflammatory NF-κB target genes in the mouse colon mucosa.** qPCR analysis of A20, ICAM1, COX-2, iNOS, MIP-2, IL1β, c-Jun and c-Fos in the proximal colon mucosa of control and sulindac-treated mice (1 week). mRNA expression was normalised to the housekeeping gene rpl19. Graphs represent relative gene expression. Error bars indicate SEM.

We previously reported that sulindac up-regulates pro-inflammatory genes in the proximal colon of mice treated with sulindac for 20 weeks [9]. These mice had developed pronounced mucosal damage and transmural inflammatory response caused by the drug. Therefore, we investigated mice treated with sulindac for 1 week in order to assess the effect of sulindac on gene expression at an early time point prior to the development of major tissue damage [9]. We selected NF-κB target genes previously implicated in colon pathogenesis [26-28], and analyzed their expression in colon mucosal tissue from control and sulindac treated mice. While the pro-inflammatory genes Cox-2, iNOS, MIP-2, IL-1β and c-Fos were significantly up-regulated by the sulindac diet (P ≤ 0.01) in the proximal colon, there was no significant change in ICAM1, A20 or c-Jun gene expression (Figure 3). This confirms the effect of sulindac on inducing pro-inflammatory gene expression *in vivo* but suggests different dynamics or selectivity of sulindac-induced NF-κB target genes *in vivo*.

### Sulindac sulfide treatment induces up-regulation of NF-κB target genes in HCT116, SW480 and SW620 cells

**Figure 4:**
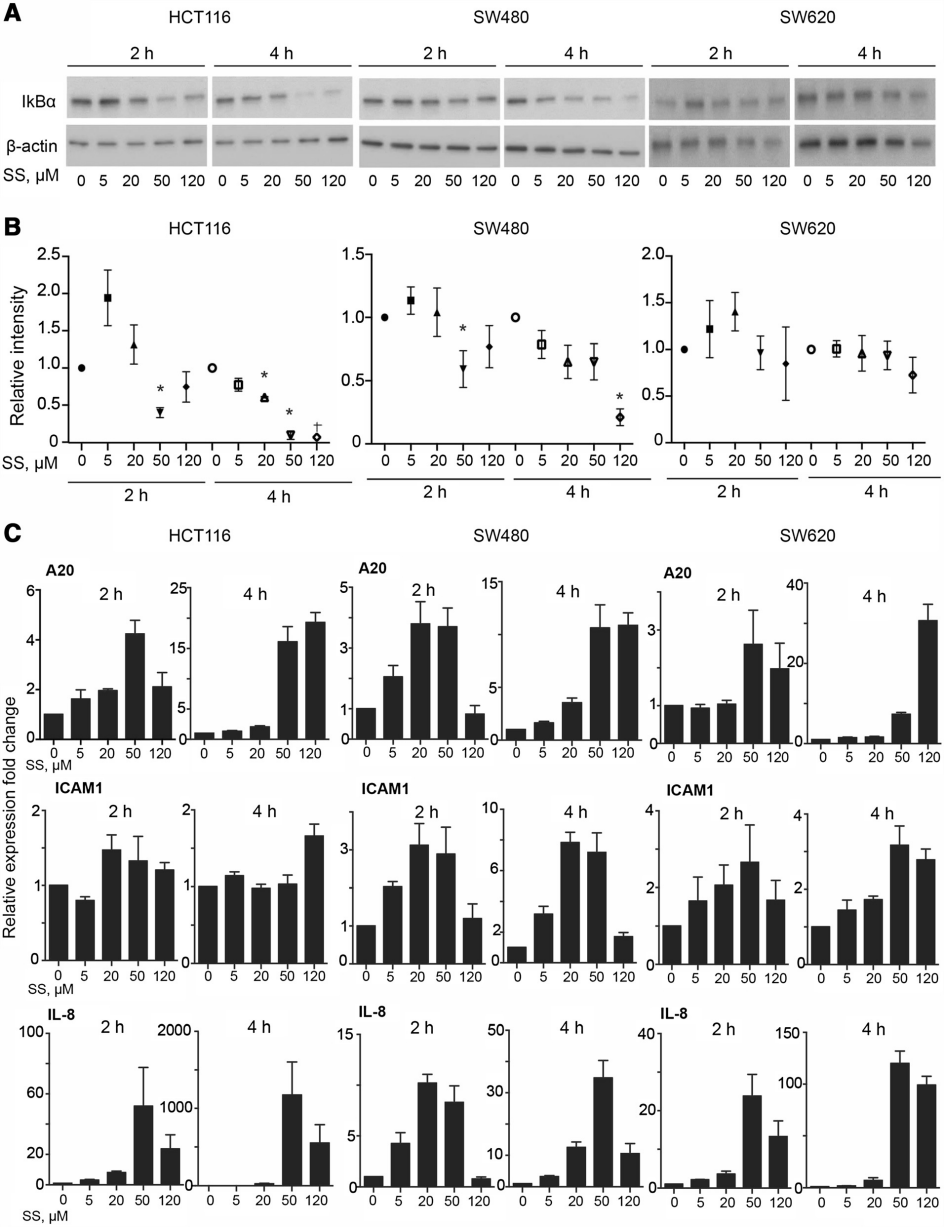
**Sulindac sulfide treatment induces up-regulation of NF-κB target genes in HCT116, SW480 and SW620 cells. (A)** HCT116, SW480 and SW620 cells were treated with the indicated doses of sulindac sulfide (SS) or the control DMSO (equivalent to the highest dose SS) for 2 and 4 hours, after which lysates were collected and western blot analysis was performed for IκBα and the loading control β-actin. **(B)** Densitometry of IκBα levels normalized to β-actin. Error bars represent SEM (n = 3). * represents P < 0.05, + average from two data points. **(C)** qPCR analysis for A20, ICAM1 and IL-8 mRNA expression. Gene expression was normalized to the house-keeping gene GAPDH. The data are presented as fold change ± SEM (from 3 independent experiments).

In order to assess whether sulindac sulfide can activate the NF-κB pathway in the background of a variety of molecular defects, we selected three additional colorectal cancer cell lines, HCT116, SW480 and SW620. NF-κB pathway activation was assessed by a western blot analysis for IκBα total levels and NF-κB target gene expression (Figure 4A, B, C). Up-regulation of NF-κB target genes A20, ICAM1 and IL-8 was observed in SW480 and SW620 cells following stimulation with sulindac sulfide but only A20 and IL-8 were strongly upregulated in HCT116 cells (Figure 4C). Furthermore, there was variation in IκBα levels following the drug treatment. The decrease in IκBα levels in response to sulindac sulfide treatment was the most pronounced in HCT116 cells but no significant changes were observed in SW620 cells. Nevertheless, mRNA levels of ICAM1 did not significantly increase in HCT116 cells except in cells treated with 120 μM sulindac sulfide for 4 hours, in contrast to the strong response seen in HCT-15, SW480 and SW620 cells. This suggests that in addition to NF-κB other factors may be modulating sulindac-induced up-regulation of pro-inflammatory cytokines.

### AP-1 as well as NF-κB transcription factors are involved in sulindac-sulfide induced activation of IL-8 gene expression

Strong up-regulation of mRNA levels for the IL-8 gene was observed in all four cell lines tested (Figures 1D, 4C). IL-8 gene expression levels are known to be strikingly variable, ranging from not detectable levels to over 100-fold activation depending on the inducing stimuli [29]. The core IL-8 promoter contains a NF-κB element as well as activating protein (AP)-1 and CAAT/enhancer-binding protein (C/EBP)-binding sites [29]. Unlike the NF-κB binding sites, the AP-1 and C/EBP sites are not essential for IL-8 induction but are required for maximum gene expression in some cell types (reviewed in [29]).

**Figure 5:**
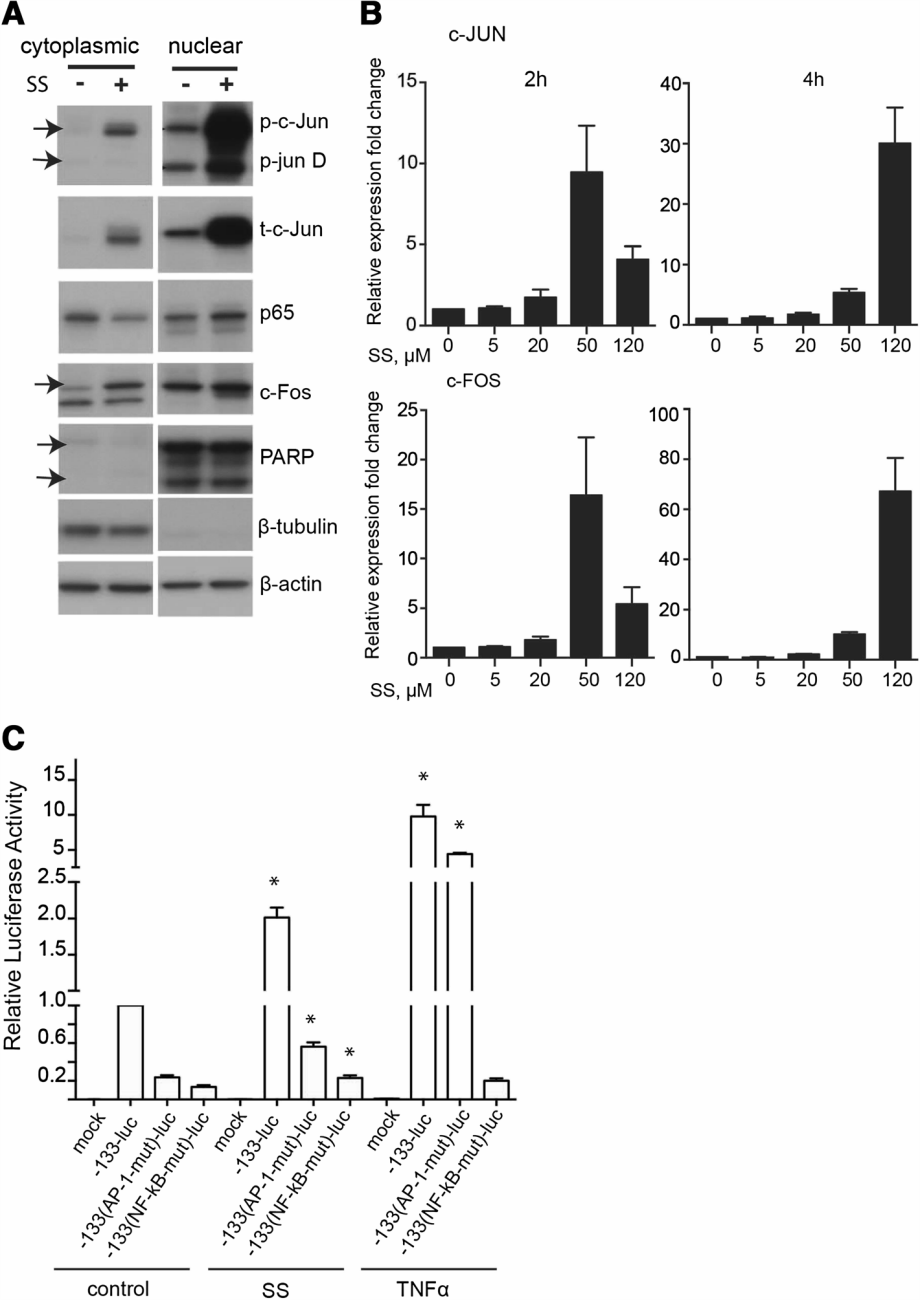
**AP-1 and NF-κB transcription factors are involved in sulindac-sulfide induced activation of IL-8 gene expression. (A)** HCT-15 cells were treated with 50 μM sulindac sulfide (SS) or the control DMSO for 4 hours. Western blot analysis of the cytoplasmic and nuclear fraction for phosphorylated c-Jun (and phosphorylated junD), c-Jun, c-Fos and p65. PARP is used as a nuclear marker while β-tubulin is used as a cytoplasmic marker. Loading control β-actin. Representative blot of two independent experiments. **(B)** qPCR analysis for c-JUN and c-FOS. Bars represent fold change of relative gene expression, normalized to the house-keeping gene GAPDH. Mean values ± SEM (from three independent experiments). **(C)** Luciferase reporter gene assay was performed to measure transcriptional activity from the human endogenous IL-8 promoter. HCT-15 cells were transfected with the IL-8 promoter reporter (-133-luc) or reporters with mutated AP-1 and NF-κB binding sites (-133(AP-1-mut)-luc and -133(NF-κB-mut)-luc respectively) and β-glactosidase reporter and treated with 20 μM sulindac sulfide (SS), the vehicle control DMSO (control) or the positive control TNFα (20 ng/ml) for 2 hours. Luciferase activity was normalized to β-galactosidase activity and is presented as mean Relative Luciferase Activity ± SEM (from three independent experiments performed in duplicate). * represents P < 0.05.

We hypothesized that the strong expression of IL-8 gene following sulindac sulfide treatment may be due to activation of both NF-κB and AP-1 transcription factors. AP-1 is a transcription factor composed of homo or heterodimers of the Jun, Fos and ATF family members [30]. We studied the nuclear and cytoplasmic protein levels of the NF-κB subunit p65 and the AP-1 members c-Fos and c-Jun in control and sulindac sulfide-treated cells (Figure 5A). Treatment with sulindac sulfide increased the amount of nuclear p65, associated with a decrease in cytoplasmic p65 levels, indicative of p65 nuclear translocation following the drug treatment (Figure 5A). The levels of total c-Jun and c-Fos were up-regulated in both the cytoplasmic and nuclear fraction upon sulindac sulfide treatment and there was a marked increase in phosphorylated c-Jun and JunD indicative of activation of the JNK pathway [31]. In consensus with the western blot results, the mRNA expression levels of c-JUN and c-FOS were markedly upregulated in the presence of 50 and 120 μM sulindac sulfide and c-FOS levels also increased following treatment with 20 μM sulindac sulfide for 4 hours (Figure 5B).

Next we assessed whether both transcription factors are required for the strong up-regulation of IL-8 gene expression using an IL-8 promoter construct cloned in a luciferase reporter vector, with or without mutated NF-κB and AP-1 binding sites (Figure 5C). Treatment with TNFα was used as a positive control and resulted in a strong increase in luciferase activity, which was slightly down-regulated in cells transfected with the AP-1 mutant binding sites but completely abolished in cells transfected with mutated NF-κB binding sites (Figure 5C). In contrast, in sulindac sulfide-treated cells both the mutated AP-1 and NF-κB binding sites strongly reduced the up-regulation in luciferase activity (P < 0.05) (Figure 5C). These results indicate that the strong up-regulation of IL-8 gene expression induced by sulindac sulfide treatment is dependent on both the NF-κB and AP-1 transcription factors, whereas TNFα up-regulates IL-8 mainly through NF-κB.

### Sulindac sulfide modulates TNFα-induced IκBα phosphorylation and degradation

Since sulindac sulfide caused a decrease in IκBα protein levels in basal conditions, and induced up-regulation of NF-κB target genes, we next tested the effect of sulindac sulfide on IκBα in conditions where the canonical NF-κB pathway is activated through stimulation by the cytokine TNFα. Upon stimulation with TNFα, NF-κB activation is preceded by rapid IκBα phosphorylation on serine 32 and 36 residues by the Inhibitor of kappa B Kinase (IKK) complex [32], leading to its proteosome-mediated degradation. We analyzed the kinetics of IκBα phosphorylation and degradation in sulindac sulfide and TNFα-treated cells.

**Figure 6:**
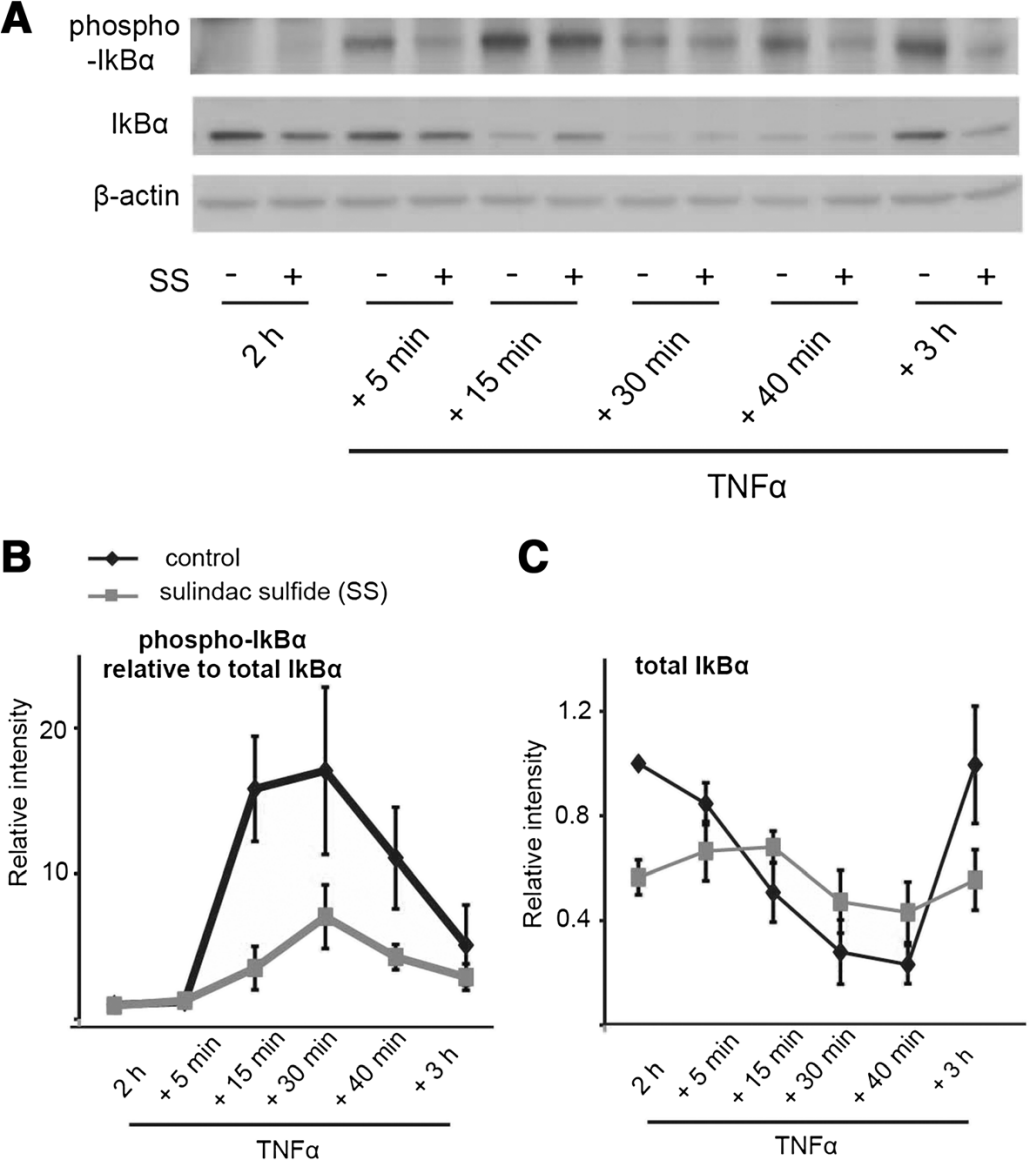
**Sulindac sulfide modulates TNFα-induced IκBα phosphorylation and degradation.** HCT-15 cells were treated with sulindac sulfide or the vehicle DMSO (control) for 2 hours. TNFα (10 ng/ml) was added to culture media for the indicated periods and cell lysates were prepared. **(A)** Western blot analysis for phosphorylated IκBα (Ser32), total IκBα and β-actin. Representative blot from four independent experiments. **(B)** Graph representing the intensity of phosphorylated IκBα, normalized to total IκBα and β-actin ± SEM. **(C)** Graph representing the intensity of IκBα, normalized to β-actin ± SEM.

HCT-15 cells were pre-treated with 50 μM sulindac sulfide for 2 hours and then stimulated with TNFα for 15 min to 3 hours. Western blot analysis of IκBα showed that sulindac sulfide pre-treatment in the absence of TNFα did not increase IκBα phosphorylation on Ser32, whereas TNFα stimulation induced rapid IκBα phosphorylation on Ser32. However, in cells pre-treated with sulindac sulfide, TNFα-induced IκBα phosphorylation was less pronounced (Figure 6A, B). TNFα caused a gradual decrease in overall IκBα protein abundance, which reached its lowest point 40 minutes after stimulation and returned to normal levels 3 hours following TNFα treatment. Sulindac sulfide pre-treatment caused a decrease in IκBα protein levels at 2 hours post-treatment and there was little change in IκBα intensity upon subsequent stimulation with TNFα (Figure 6A, C).

In summary, sulindac sulfide treatment reduced the total levels of IκBα in unstimulated cells, suggesting activation of the NF-κB pathway, but with a slower kinetics compared to TNFα. In contrast, sulindac sulfide treatment appeared to inhibit TNFα-induced phosphorylation on IκBα (Ser 32) which is consistent with previous studies [14]. These results imply that sulindac sulfide inhibits TNFα-induced NF-κB activation at a level upstream of IκBα degradation.

### Sulindac sulfide promotes up-regulation of the NF-κB target gene A20

**Figure 7:**
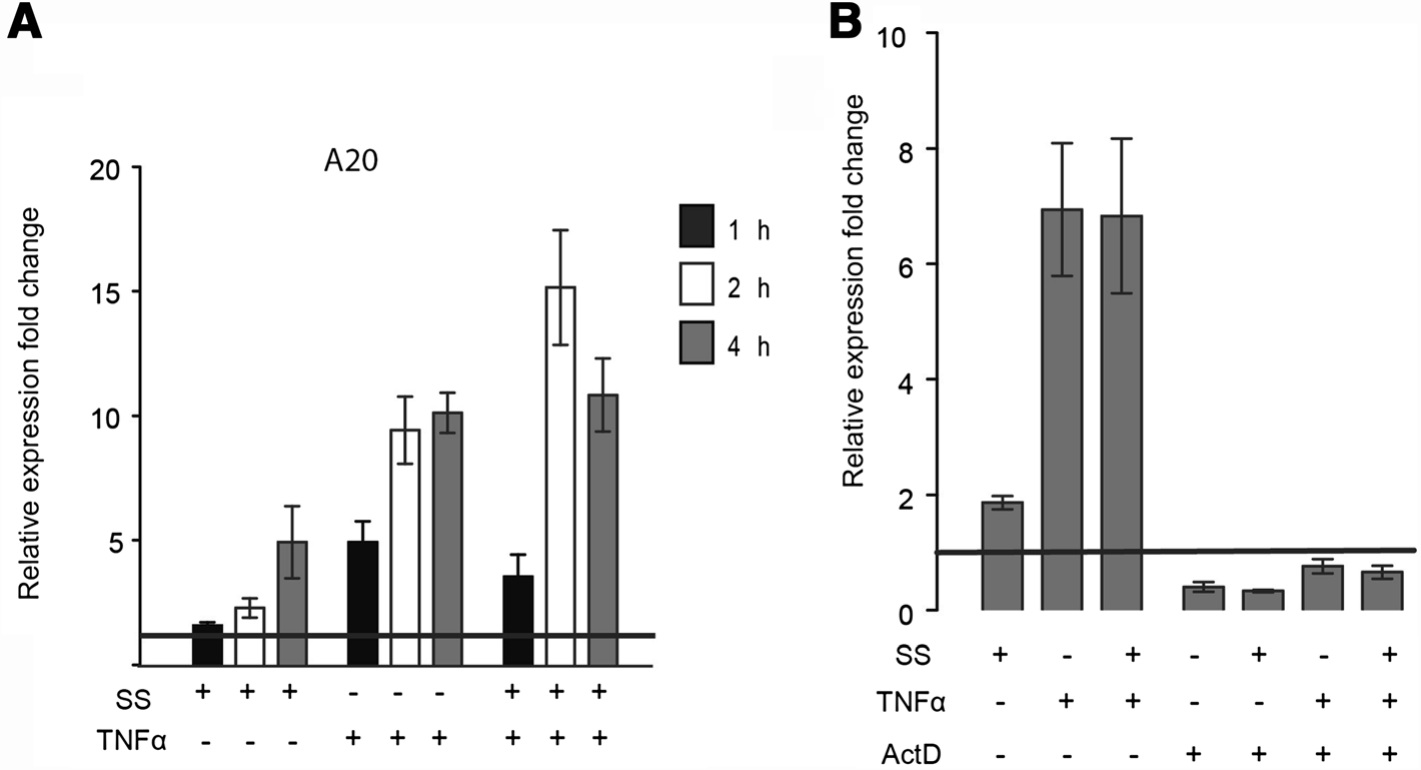
**Sulindac sulfide induces transcriptionally-dependent up-regulation of A20 mRNA levels.** qPCR analysis for A20 mRNA expression. HCT-15 cells were treated with 50 μM sulindac sulfide (SS), the vehicle DMSO (control line) or 10 ng/ml TNFα for the indicated time points. A20 gene expression was normalized to the house-keeping gene GAPDH. The data are presented as fold change ± SEM (from 4 to 7 independent experiments). **(A)** Cells were treated with the control, SS, TNFα or both in combination for the indicated time points. **(B)** Cells were pre-treated with 1 μM actinomycin D (ActD) and then treated with the indicated compounds for 4 hours.

To further explore the effects of sulindac sulfide on the NF-κB pathway in basal and TNFα stimulated cells, we studied the expression of the early response NF-κB target gene *TNFAIP3* (A20), which is not known to be targeted by any other transcription factor. NF-κB activation is necessary for A20 transcription as IKK deficiency abolishes TNFα-induced A20 transcription [20,21]. HCT-15 cells were treated with sulindac sulfide alone, TNFα alone, or both compounds in combination for 1 to 4 hours (Figure 7A). Both sulindac sulfide and TNFα, as well as the combination of the two, increased A20 mRNA levels compared to cells treated with the control. The combination of sulindac sulfide and TNFα did not result in a sustained increase in A20 mRNA levels more than that of TNFα treatment alone (Figure 7A). Taken together these results imply that sulindac sulfide does not synergise with TNFα or inhibit TNFα-induced A20 mRNA expression.

In order to test whether sulindac sulfide-induced A20 up-regulation is transcriptionally dependent, cells were pre-treated with the transcription inhibitor actinomycin D. As expected actinomycin D reduced A20 mRNA expression in cells stimulated with TNFα, confirming that the selected dose of 1 μM actinomycin D inhibits gene transcription. Sulindac sulfide also failed to up-regulate A20 mRNA expression in the presence of actinomycin D compared to vehicle control cells (Figure 7B). This result is consistent with a mechanism of sulindac sulfide-induced up-regulation of A20 mRNA that is dependent on transcriptional activation.

## Discussion

The NSAID sulindac has shown promising potential in colon cancer chemoprevention. However, serious concerns about gastrointestinal and cardiovascular side effects, including colon inflammation, perforation and bleeding, limit the clinical use of NSAIDs. We recently reported that long-term use of dietary sulindac can cause localized inflammation in the mouse proximal colon and that the inflammatory lesions are characterized by expression of pro-inflammatory NF-kB target genes [9]. This led us to explore the molecular effects of sulindac sulfide on the NF-κB pathway *in vitro*, a pathway implicated in both inflammation and malignancy. This study shows for the first time that sulindac sulfide can induce NF-kB and AP-1 mediated pro-inflammatory gene expression as well as trigger cancer cell death in the same experimental conditions. These findings may have implications for understanding the mechanism of NSAID-induced colon damage and inflammation.

Sulindac sulfide-induced up-regulation of NF-κB target genes was detected in four colorectal cancer cell lines, HCT-15, HCT-116, SW480 and SW620 as well as in the mucosa of mouse proximal colon one week after the start of sulindac diet. Sulindac sulfide treatment also resulted in transcriptional and translational up-regulation of the AP-1 transcription factor components c-FOS and c-JUN, accompanied by an increase in nuclear accumulation of p65, c-Fos and c-Jun.

The strongest up-regulation was seen for the chemokine IL-8, both *in vivo* (the murine homologue MIP-2) and *in vitro* (IL-8) [9]. IL-8 plays a key role in promoting proliferation and survival of endothelial and cancer cells, angiogenesis and neutrophil infiltration [11,33]. IL-8 was the single most differentially expressed gene among 6000 significantly expressed genes in gastric epithelial cell line in response to *Helicobacter pylori* exposure [34]. Cooperation between AP-1 and NF-κB is required for optimal IL-8 gene induction in virus infected airway epithelium [35]. In order to assess whether NF-κB and AP-1 cooperation was required for the up-regulation of IL-8 mRNA levels in HCT-15 cells, we used the IL-8 promoter element cloned into a luciferase reporter construct with wild type or mutated NF-κB and AP-1 binding sites. Mutation of either NF-κB or AP-1 binding sites diminished the luciferase activity upon sulindac sulfide stimulation, whereas mutation of the AP-1 binding site had less effect after TNFα stimulation. These results indicate that the strong up-regulation of IL-8 gene expression induced by sulindac sulfide treatment is dependent on both the NF-κB and AP-1 transcription factors, whereas TNFα up-regulates IL-8 mainly through NF-κB. In agreement with this study, it has been reported that treatment of acute myeloid leukemia (AML) cell lines or bone marrow cells from AML patients with sulindac sulfide increases the expression of AP-1 family transcription factors [31]. AP-1 transcription factor is involved in the regulation of many cytokine genes and is therefore an important component of the inflammatory response [36]. Furthermore it has been suggested that NF-κB can modulate AP-1 activity, raising the intriguing possibility that the interplay by the two transcription factors can fine-tune the inflammatory response [37].

Treatment of mice for 1 week with sulindac also resulted in significant up-regulation of other NF-κB target genes IL-1β, iNOS and Cox-2 as well as c-Fos in the colon mucosa. This suggests that sulindac induces an early strong immunogenic response in the mouse colon. However, in contrast to sulindac sulfide treatment *in vitro* there was no significant change in ICAM-1 or A20 gene expression. This is consistent with our previous findings in mice treated with sulindac for 20 weeks [9], Although A20 is a target of NF-κB, it is actually suppressed in inflammatory bowel disorders, where NF-κB is activated. A20 is a part of the negative feedback loop of NF-κB signaling and A20 deregulation in inflammatory disorders is consistent with its role as an anti-inflammatory factor [38]. For example, A20 is down-regulated in Crohn’s disease and enterocyte-specific deficiency of A20 in mice results in increased susceptibility to experimental colitis [28,39]. This highlights the complex microenvironment in the colon inflamed tissue, where cross-talk between inflammatory and colon epithelial cells further modulates gene expression.

NF-κB also regulates the transcription of TNFα and we have previously shown that sulindac sulfide induces TNFα expression [9]. It is possible that sulindac sulfide-induced TNFα acts in an autocrine manner to activate the NF-κB pathway. TNFα-mediated NF-κB activation can lead to cell survival or cell death depending on the metabolic context of the cell and the contribution of other signalling pathways such as AKT activation and the JNK signalling cascade [40-42]. Although TNFα was initially associated with tumour necrosis [43], mounting evidence suggests that TNFα plays a role in tumour growth and progression [44], induction of genes involved in inflammation, tissue repair [45] and angiogenesis [46]. Thus sulindac sulfide-induced activation of the NF-κB pathway may result in a positive feedback loop through induction of TNFα and may contribute to the pro-inflammatory or anti-tumorigenic activity of the drug depending on the cellular context.

NF-κB is known to exert pro-survival signals, but here we show that sulindac sulfide induces cell death at a concentration that also activates NF-κB. The most prominent activation of NF-κB target genes was seen with concentration (50 μM) of the drug that left the majority of cells viable. In some conditions molecules released during the apoptotic response can activate the NF-κB pathway. However, here sulindac sulfide treatment resulted in NF-κB activation in the presence or absence of the pan-caspase inhibitor Q-VD-Oph, which effectively inhibits apoptosis, indicating that the drug-induced NF-κB activity is not a consequence of sulindac-induced apoptosis. Our experiments do not exclude the involvement of NF-κB in sulindac sulfide-induced apoptosis. Under certain stimuli NF-κB activity may lead to cell death [47] but sulindac also activates NF-κB-independent pro-apoptotic pathways. Interestingly, suppression of NF-κB activity with the inhibitor PDTC potentiated sulindac sulfide-induced cell death by necrosis. PDTC use in the clinic for the treatment of colon cancer has been supported by a number of studies and there are reports of PDTC enhancing the anti-cancer potential of 5-fluorouracil (5-FU) [48]. As PDTC potentiates sulindac sulfide-induced cancer cell death while inhibiting sulindac-sulfide-induced up-regulation of pro-inflammatory factors, it is possible that the combination of PDTC and sulindac may be further explored as a relevant therapeutic option for colorectal cancer.

A previous report has suggested that sulindac sulfide inhibits the NF-κB pathway but the experimental conditions were different from our study and IL-8, A20 and ICAM1 gene expression was not analyzed [14]. Sulindac sulfide inhibited NF-κB-inducible kinase (NIK)-induced IKKβ kinase activity in COS cells, but at a concentration that was four times higher than used here. In the same study this inhibition was not seen with the 40 μM concentration of sulindac sulfide [14]. IKKβ is an essential kinase upstream of IκBα, crucial for the activation of NF-κB through the canonical pathway. Also, in another study sulindac sulfide administered at doses higher than 50 μM inhibited IKKβ activity and NF-κB DNA binding activity in HCT116 colon cancer cells [49]. This implies that the inhibitory effect of sulindac sulfide on the NF-κB pathway may be concentration dependent.

Although this study is the first to report an activating role of the NSAID sulindac sulfide on the NF-κB pathway, celecoxib, a COX-2 selective NSAID was previously shown to have similar effects on NF-κB signaling both *in vitro* and *in vivo.* At 50 μM celecoxib resulted in an increase in IL-1β-induced NF-κB DNA binding activity and NF-κB-dependent gene expression, although in another study celecoxib was found to have an inhibitory role on NF-κB activity [15,50]. Similarly, the COX-2 selective NSAID NS-398 induced an increase in NF-κB DNA binding activity but not in NF-κB-reporter gene expression in colon cancer cells while indomethacin, a drug closely related to sulindac, was reported to induce gastropathy through activation of NF-κB in gastric microvascular endothelial cells [51,52]. Further studies are necessary to determine if other NSAIDs activate the NF-κB pathway.

## Conclusions

In summary, this study provides experimental evidence that the pharmacologically-active sulindac metabolite, sulindac sulfide, activates NF-κB-mediated endogenous gene transcription in colon cells, including NF-κB target pro-inflammatory factors *in vitro* and *in vivo*. This is the first report to show that sulindac sulfide activates both NF-κB and AP-1 transcription factors, which may be important in NSAID-induced gastrointestinal toxicity [53-56] and the increased risk of acute myocardial infarction in patients receiving some NSAIDs [57]. These results imply that some of the adverse effects caused by sulindac in the mouse colon such as inflammation and ulceration may be caused by aberrant immunoregulation in the colon mucosa. Further studies are required to address sulindac activation of NF-κB *in vivo* and whether this is responsible for the side effects of NSAIDs in the human colon.

## Methods

### Tissue culture and reagents

HCT-15, HCT116 and SW620 cells (CCL-225, CCL-247, CCL-227; ATCC, USA) were propagated in RPMI 1640 (GIBCO®, Invitrogen Corporation) supplemented with 10% fetal bovine serum (FBS) (Hyclone Laboratories Inc., Australia), HEPES (10 mM, GIBCO®), glutamine (4 mM, GIBCO®), insulin (10 μg/ml, Actrapid, Novo Nordisk, Australia) and gentamycin (20 μg/ml, Pfizer, Australia), except as noted. SW620 cells were propagated in RPMI 1640 (GIBCO®) with 10% FBS and 20 μg/ml gentamycin. For experiments cells were plated at 2×10^5^ cells/well in 6-well culture plate (Corning) and cells were incubated overnight in reduced serum conditions (0.2% FBS) prior to treatment with the indicated reagents. The cell lines were authenticated by CellBank Australia in 2011 using an Identifiler PCR Amplification Kit (Applied Biosystems, USA). Tumor necrosis factor α (TNFα) was obtained from Peprotech Inc., (USA); sulindac sulfide, PDTC, actinomycin D and DMSO from Sigma-Aldrich (MO, USA); Q-VD-OPh from MP Biomedicals (USA). Sulindac sulfide, actinomycin D and Q-VD-OPh were dissolved in DMSO while PDTC was dissolved in distilled water.

### Mice and sulindac diet

Mice on the C57Bl/6J background were bred in specific pathogen free conditions. Mice (6 weeks old) were given a diet containing 320 p.p.m. sulindac (Sigma-Aldrich; Specialty Feeds, Australia) for 1 week or control feed ad libitum. The diet was standard mouse cubes. This study was carried out in accordance with the recommendations of the National Health and Medical Research Council (Australian Code of Practice for the Care and Use of Animals for Scientific Purposes). All animal experiments were approved by the Garvan Institute of Medical Research Animal Ethics Committee (protocol no. 10/40).

### mRNA and protein analysis

The mucosal surface of the proximal colonic tissue was lightly scraped and snap frozen in liquid nitrogen for RNA extraction. RNA from mouse tissue or cell lines was extracted using Qiagen RNeasy mini (QIAGEN GmbH, Germany). Q-PCR reactions were performed using SYBRgreen, (Applied Biosystems, USA), Taqman (Applied Biosystems, USA) or UPL assays (Roche Applied Science) on ABI Prism 7900-HT Real Time PCR system (Applied Biosystems) or the Roche Lightcycler 480 (Roche Applied Science). For protein analysis cell lysates [58] were separated on polyacrylamide gels (10-15%) and transferred to polyvinylidene difluoride (PVDF) membranes. The membranes were blocked with 5% (w/v) bovine serum albumin, dissolved in 0.2% (v/v) Tween20/ tris-buffered saline (TBS). The membranes were incubated with primary antibodies for 1 h RT or overnight at 4°C (β-actin, 1:10 000, clone AC15, Sigma; IκBα #9242, 1:1000; Phospho-IκBα (Ser32) #2859 14D4; cleaved caspase 3 (Asp175) #9661, Phospho-c-Jun (Ser73) #9164, c-Jun #9165 (Cell Signalling Technology Inc., USA), c-Fos ab7963, beta-tubulin ab6046 (Abcam, UK), p65 (A) sc-109 (Santa Cruz Biotechnology Inc., USA). ImageJ densitometry software (National Institutes of Health, USA) or Quantity One software (Bio-Rad Laboratories, Canada) were used for gel band quantitative densitometric analysis.

### Nuclear/cytoplasmic fractionation

Cells were plated at 32.575×10^5^ cells in 150x20 mm Petri dish (Corning) and cells were incubated overnight in reduced serum conditions (0.2% FBS) prior to treatment with sulindac sulfide. Cells were lysed with Cayman nuclear extraction kit No10009277 (Cayman Chemicals, USA) according to the manufacturer’s instructions. Lysates were resolved on 10% polyacrylamide gels and transferred to polyvinylidene difluoride (PVDF) membranes.

### P65(RelA) DNA-binding assay

P65 binding was assessed using Cayman's p65 transcription factor assay (Cayman Chemicals, USA). A double-stranded oligonucleotide that contained a consensus p65 binding site was immobilized in all plate wells and incubated with previously prepared flash frozen nuclear extracts (7.5 μg/well) overnight at 4°C without shaking. The plate was washed extensively according to manufacturer’s instructions and incubated with a primary anti-p65 antibody, followed by a secondary antibody conjugated with horseradish peroxidase (HRP) that was used for detection. The absorbance is expressed as the optical density at 450 nm, normalized to the background readings. Positive and negative controls were included in the assay kit.

### Detection of apoptosis

#### ***Trypan blue exclusion assay***

After the indicated treatments, cells floating in the media and trypsinized adherent cells were collected. Cells were incubated in 1:1 ratio with 0.4% Trypan blue (GIBCO) and were counted under a phase contrast microscope or using the Countess™ automated cell counter (Invitrogen). Cells with compromised membrane integrity are positive for trypan blue and were represented as a percentage of total counted cells.

#### ***Flow cytometry analysis for AnnexinV/propidium iodide***

Apoptosis was detected by dual staining for phosphatidylserine (PS) externalization (AnnexinV-fluorescent isothiocyanate-conjugated Fluos) and propidium iodide (PI) cell incorporation by flow cytometry (BD FACSCanto™ flow cytometer) using the Annexin-V-Fluos staining kit (Roche Applied Science) according to the manufacturer’s instructions. Briefly, after treatment and trypsinization, adherent and detached cells from different treatment groups were counted and incubated for 15 min at 15–25°C with Annexin-V-Fluos labeling solution. PS externalization is a specific marker of early apoptotic events while PI is taken only by cells with compromised cell membrane (necrosis). Cells were considered apoptotic when they were Annexin-V-positive and PI-negative and necrotic when they were Annexin-V- and PI- positive or Annexin-V-negative and PI-positive. Appropriate electronic compensation of the instrument was used in order to exclude overlapping of the two emission spectra.

### Luciferase reporter assay for IL-8 expression

Cells (2×10^5^/well) were plated into a 6-well plate, in duplicate for each treatment group. Transfection for every well was performed with 0.1 μg β-galactosidase internal control plasmid (pEF-DEST51lacZ) and 0.8 μg IL-8 promoter luciferase reporter constructs [59]. Transfection was performed in 10% FCS media, using 3 μl/well X-tremeGene HP DNA transfection reagent (Roche) following the manufacturer’s instructions. After stimulation, adherent cells were lysed in lysis buffer (Galacto-Star™ System, T2071-0607049, Tropix®, USA) on ice. The supernatant was used for the luciferase assay. Samples (from each well of cultured cells) were pipetted in duplicate into a 96-well luminometer plate (OptiPlate™-96, 6005290, PerkinElmer®, Zaventem, Belgium). β-galactosidase activity was determined by addition of freshly diluted Galacto-Star™ reagent, following an incubation for 30 min at RT. Plates were read using the FLUOstar OPTIMA microplate reader (BMG LABTECH GmbH, Germany) with a luminescence optic reader configuration and automatic reagent injection. Luciferase activity was determined by addition of freshly diluted luciferine (Luciferase Assay System, Cat #E1501, Promega, USA) to the lysates and the plates were read immediately as above. Control for transfection efficiency in each well from the multi-well culture plate was obtained by assessing the β-galactosidase activity of the lysate for that well. Relative luciferase activity for a sample was determined by dividing the average luciferase activity by the relative amount of β-galactosidase activity.

The reporter constructs were a kind gift by Professor Naofumi Mukaida, Cancer Research Institute, Kanazawa University Kakuma-machi, Japan [59]. The constructs used contained the 5’ region of the IL-8 gene spanning downstream from -133 bp with wild type or mutated AP-1 and NF-κB sites. This region contains three cis elements, AP-1 (2126 to 2120 bp), NF–IL-6-like (member of the C/EBP family) (294 to 281 bp), and κB-like (280 to 270 bp) sites [59]. The constructs were named: -133-luc; -133(NF-κB-mut)-luc; -133(AP-1-mut)-luc.

### Statistical analysis

The data are graphed as mean ± SEM from at least 3 independent experiments. Student t test was used for comparisons between two groups. A P value of <0.05 was considered significant.

## Abbreviations

A20: Zinc finger protein A20; AP-1: Activator protein-1; COX: Cyclooxygenase; HMGB1: High mobility group box 1 protein; ICAM1: Intercellular adhesion molecule 1; IκBα: Inhibitor of kappa B alpha; IKK: I kappa B kinase; IL-8: Interleukin 8; MIP-2: Macrophage inflammatory protein 2; NF-κB: Nuclear factor kappa B; NSAID: Non-steroidal anti-inflammatory drug; PDTC: Pyrrolidine dithiocarbamate; Q-VD-OPh: (3S)-5-(2,6-Difluorophenoxy)-3-[[(2S)-3-methyl-1-oxo-2-[(2-quinolinylcarbonyl)amino]butyl]amino]-4-oxo-pentanoic acid hydrate; SS: sulindac sulfide; TNFα: Tumor necrosis factor alpha.

## Competing interests

The authors declare that they have no competing interests.

## Authors’ contributions

DM designed and conducted most experiments, analyzed the data and wrote the manuscript. DM, MKC and SG conceived the project and wrote the manuscript. NC, LP and IN designed and conducted experiments, and analyzed the data. EAM provided valuable advice throughout the study and helped writing the manuscript. All authors read and approved the final version of the manuscript.
